# Direct attenuation correction for ^99m^Tc-3PRGD_2_ chest SPECT lung cancer images using deep learning

**DOI:** 10.3389/fonc.2023.1165664

**Published:** 2023-05-12

**Authors:** Haiqun Xing, Tong Wang, Xiaona Jin, Jian Tian, Jiantao Ba, Hongli Jing, Fang Li

**Affiliations:** Department of Nuclear Medicine, Peking Union Medical College Hospital, Chinese Academy of Medical Science and Peking Union Medical College, Beijing Key Laboratory of Molecular Targeted Diagnosis and Therapy in Nuclear Medicine, Beijing, China

**Keywords:** ^99m^Tc-3PRGD2, Chest SPECT, CT-attenuation correction, deep learning attenuation correction, lung cancer

## Abstract

**Introduction:**

The attenuation correction technique of single photon emission computed tomography (SPECT) images is essential for early diagnosis, therapeutic evaluation, and pharmacokinetic studies of lung cancer. ^99m^Tc-3PRGD_2_ is a novel radiotracer for the early diagnosis and evaluation of treatment effects of lung cancer. This study preliminary discusses the deep learning method to directly correct the attenuation of ^99m^Tc-3PRGD_2_ chest SPECT images.

**Methods:**

Retrospective analysis was performed on 53 patients with pathological diagnosis of lung cancer who received ^99m^Tc-3PRGD_2_ chest SPECT/CT. All patients’ SPECT/CT images were reconstructed with CT attenuation correction (CT-AC) and without attenuation correction (NAC). The CT-AC image was used as the reference standard (Ground Truth) to train the attenuation correction (DL-AC) SPECT image model using deep learning. A total of 48 of 53 cases were divided randomly into the training set, the remaining 5 were divided into the testing set. Using 3D Unet neural network, the mean square error loss function (MSELoss) of 0.0001 was selected. A testing set is used to evaluate the model quality, using the SPECT image quality evaluation and quantitative analysis of lung lesions tumor-to-background (T/B).

**Results:**

SPECT imaging quality metrics between DL-AC and CT-AC including mean absolute error (MAE), mean-square error (MSE), peak signal-to-noise ratio (PSNR), structural similarity (SSIM), normalized root mean square error (NRMSE), and normalized Mutual Information (NMI) of the testing set are 2.62 ± 0.45, 58.5 ± 14.85, 45.67 ± 2.80, 0.82 ± 0.02, 0.07 ± 0.04, and 1.58 ± 0.06, respectively. These results indicate PSNR > 42, SSIM > 0.8, and NRMSE < 0.11. Lung lesions T/B (maximum) of CT-AC and DL-AC groups are 4.36 ± 3.52 and 4.33 ± 3.09, respectively (p = 0.81). There are no significant differences between two attenuation correction methods.

**Conclusion:**

Our preliminary research results indicate that using the DL-AC method to directly correct ^99m^Tc-3PRGD_2_ chest SPECT images is highly accurate and feasible for SPECT without configuration with CT or treatment effect evaluation using multiple SPECT/CT scans.

## Introduction

Lung cancer is one of the most commonly diagnosed cancers and the leading cause of cancer-related deaths worldwide with an estimated two million new cases each year. The incidence and mortality of lung cancer in China accounted for 37.0% and 39.8% of the world’s total, respectively, ranking first in cancer incidence and mortality (1, 2). Non-small cell lung cancer (NSCLC) accounts for almost 85% of lung cancer (3). Early diagnosis and accurate staging are essential to improve the survival rate of lung cancer patients. With the deepening of the research on the molecular pathological mechanism of lung cancer, new biomarkers have been discovered and used for clinical diagnosis and targeted therapy. Molecular imaging can non-invasively detect dynamic molecular processes *in vivo*, offering enormous potential for early diagnosis, accurate staging, and guidance for treatment. Accordingly, molecular imaging agents would be highly useful in the non-invasive detection of lung cancer.

The arginine–glycine–aspartic acid (RGD) tripeptide sequence can specifically bind to the integrin α_V_β_3_ receptors, which is highly expressed on tumor cells and activated endothelial cells. ^99m^Tc-PEG_4_-E[PEG_4_-c(RGDfK)]_2_ (^99m^Tc-3PRGD_2_) is a novel RGD containing single photon emission computed tomography (SPECT) radiotracer targeting integrin α_V_β_3_ receptors for tumor detection, angiogenesis imaging, and tumor therapy efficacy assessment. Preliminary results from multicenter studies indicate that ^99m^Tc-3PRGD_2_ imaging is sensitive for the detection of lung malignancies (4, 5). Attenuation correction of ^99m^Tc-3PRGD_2_ SPECT chest tomography images is the basis of accurate quantification. To date, some SPECT devices have been configured with the co-location CT, commonly known as SPECT/CT, to perform attenuation correction of SPECT images. However, there are still some SPECT devices that are not configured with CT, and the adopted off-machine CT attenuation correction or uniform image attenuation correction is subject to large errors. Even for patients using SPECT/CT for treatment monitoring, it is not necessary to use CT scan images for attenuation correction of SPECT in every SPECT/CT scan to reduce the CT radiation dose of patients. SPECT attenuation correction has received attention in clinical practice.

The deep learning method has been used to directly perform attenuation correction studies on PET and SPECT images, and satisfactory results have been obtained for attenuation correction of myocardial perfusion SPECT images (6, 7). This can improve the attenuation correction accuracy of the SPECT/CT images and provide a new accurate and efficient attenuation correction method for SPECT without CT. At present, there are no studies involving deep-learning-based attenuation correction studies of ^99m^Tc-3PRGD_2_ for chest SPECT images. In this study, the deep-learning-based attenuation correction method for ^99m^Tc-3PRGD_2_ chest SPECT images of lung cancer patients is discussed.

## Materials and methods

This retrospective study was approved by the ethics committee of Chinese Academy of Medical Sciences and Peking Union Medical College Hospital. From 2012 to 2017, 53 patients with pathological diagnoses of lung cancer were collected from ^99m^Tc-3PRGD_2_ chest SPECT images using the Precedence SPECT/CT system (Philips, Netherlands). Written informed consent was obtained from each patient. All patients underwent ^99m^Tc-3PRGD_2_ chest SPECT/CT. The 53 patients included 26 women and 27 men, aged 38–72 years (mean age ± SD, 60.40 ± 7.21 years old). The maximum diameter of lung lesions ranged from 1.60 to 106.60 mm (mean ± SD, 39.94 ± 20.25 mm). Chest SPECT scans were acquired 1 h after intravenous injection of ^99m^Tc-3PRGD_2_ at a dose of 11.1 MBq/kg. SPECT scan parameters were as follows: low-energy high-resolution collimators and energy window of 140 keV ± 7.5%, 360° scan, 64 projections, per projection of 30 s, zoom of 1.0, matrix of 128 × 128, and pixel of 4.664 mm. A low-dose chest scan CT was used to correct the attenuation of SPECT images. CT parameters were as follows: 120 kVp/30 mAs, matrix of 512 × 512, pixel of 0.68 mm, and thickness of 3.0 mm. After scanning, the SPECT projection data and the CT reconstruction image were transferred to the image processing workstation. SPECT attenuation correction images (CT attenuation correction, CT-AC) and the images without attenuation correction (non-attenuation correction, NAC) were obtained throughout the image processing.

A total of 53 cases with ^99m^Tc-3PRGD_2_ chest SPECT images (CT-AC and NAC) were divided into a training set (n = 48; maximum lung lesions diameter, 39.98 ± 20.94 mm) and a testing set (n = 5; maximum lung lesions diameter, 39.52 ± 13.39 mm). CT-AC SPECT images were used as the reference standard (Ground Truth) to compare SPECT images with deep learning attenuation correction (DL-AC).

### Training set and testing set images

**Figure 1:**
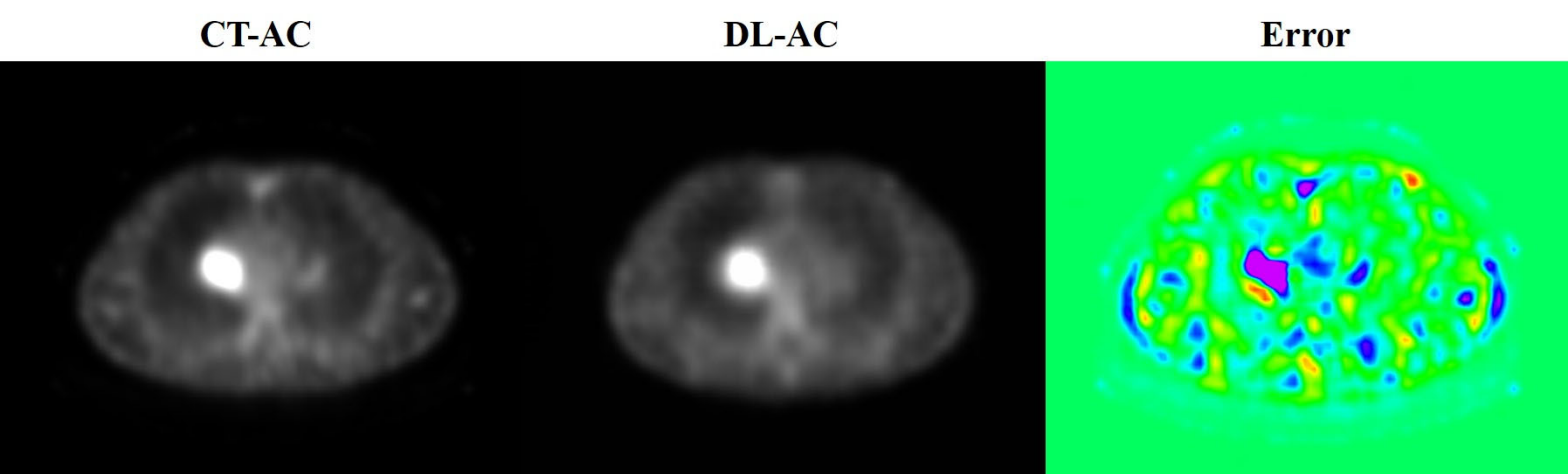
3D Unet framework used for build modeling. The 3D grayscale image is used as input, and the output image size is (128, 128, 128).

Through the MONAI deep learning framework, the depth of neural network using 3D Unet is shown in **Figure 1**. The training set was pre-processed by uniformly adjusting the image size to (128, 128, 128). The data augmentation was done by randomly rotating 90°. The parameters of the 3D Unet network in this study are as follows:

- The number of network input channels is 1; that is, the input is a 3D grayscale map.
- The number of network output channels is also 1, and the output image size is (128, 128, 128).
- After each convolution operation in the network encoder part, the number of output channels is successively (16, 32, 64, 128, 256), and the stride is 2.
- The number of residual units is 3.
- The normalization method is batch normalization.

The mean square error loss function (MSELoss) was selected as 0.0001 in this study. Five testing sets were used to evaluate the trained DL-AC model.

### Image quality evaluation and statistical analysis of data

The DL-AC effect was analyzed using both measurements of the ^99m^ Tc-3PRGD_2_ whole SPECT chest images and local lesion method.

**Figure 2:**
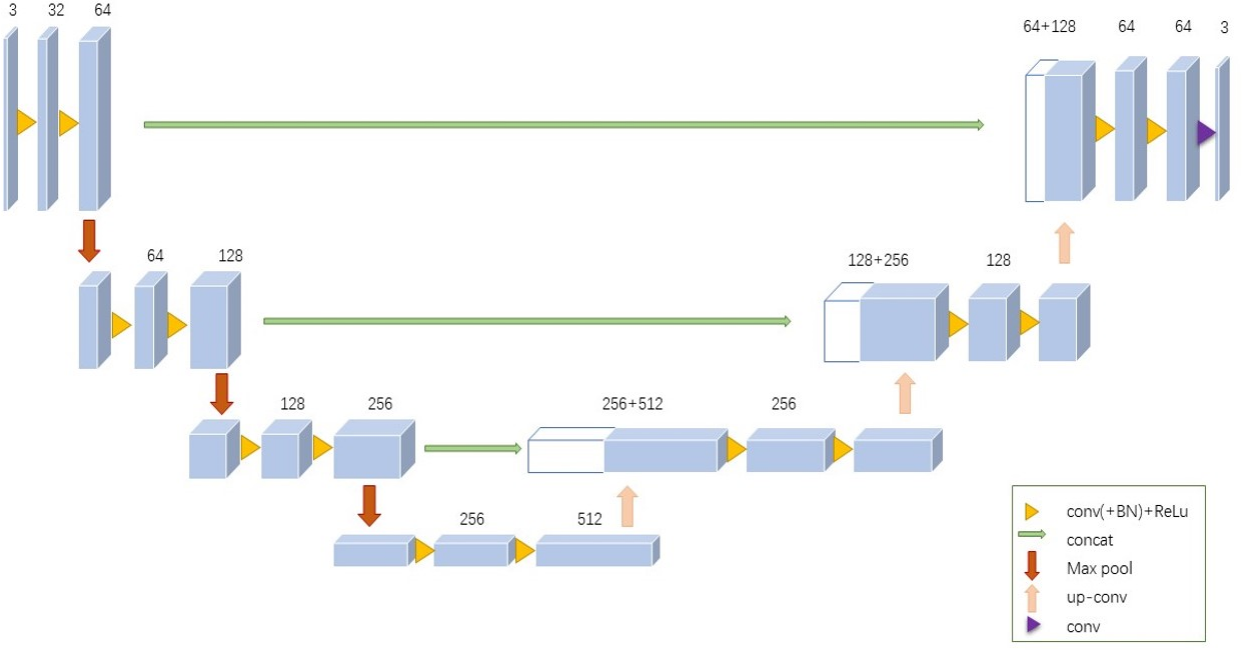
Outline of lung lesion and background. Tumor indicate lung lesions that are drawn with a preset threshold of 42%. The background is a mirror ROI set over the contralateral normal lung as a control.

1) The error and similarity metrics between the SPECT attenuation correction with CT-AC and DL-AC are to be calculated. The evaluation indexes used by referring to the reported methods (8) include mean absolute error (MAE), mean square error (MSE), peak signal-to-noise ratio (PSNR), structural similarity (SSIM), normalized root mean square error (NRMSE), and normalized mutual information (NMI). The error was expressed as mean ± SD.
2) T/B ratios of pulmonary lesions under SPECT CT-AC and DL-AC were measured. The tumor region of interest (ROI) was drawn by a preset threshold of 42% and was adjusted minimally according to visual assessment. Then, a mirror ROI was set over the contralateral normal lung as a control (**Figure 2**). The T/B ratios were calculated by (maximum and mean counts of the tumor ROI)/(maximum and mean counts of the control ROI). In cases where more than one lesion was detected in the lung, the lesion with the highest uptake was used for analysis. The T/B ratios were expressed as mean ± SD.

All the statistical analysis in this study were performed using R 3.5.1 and Python 3.5.6. A self-paired *t-*test was used to compare the T/B ratios obtained from the same patients. p < 0.05 indicated statistical significance.

## Results

**Figure 3:**
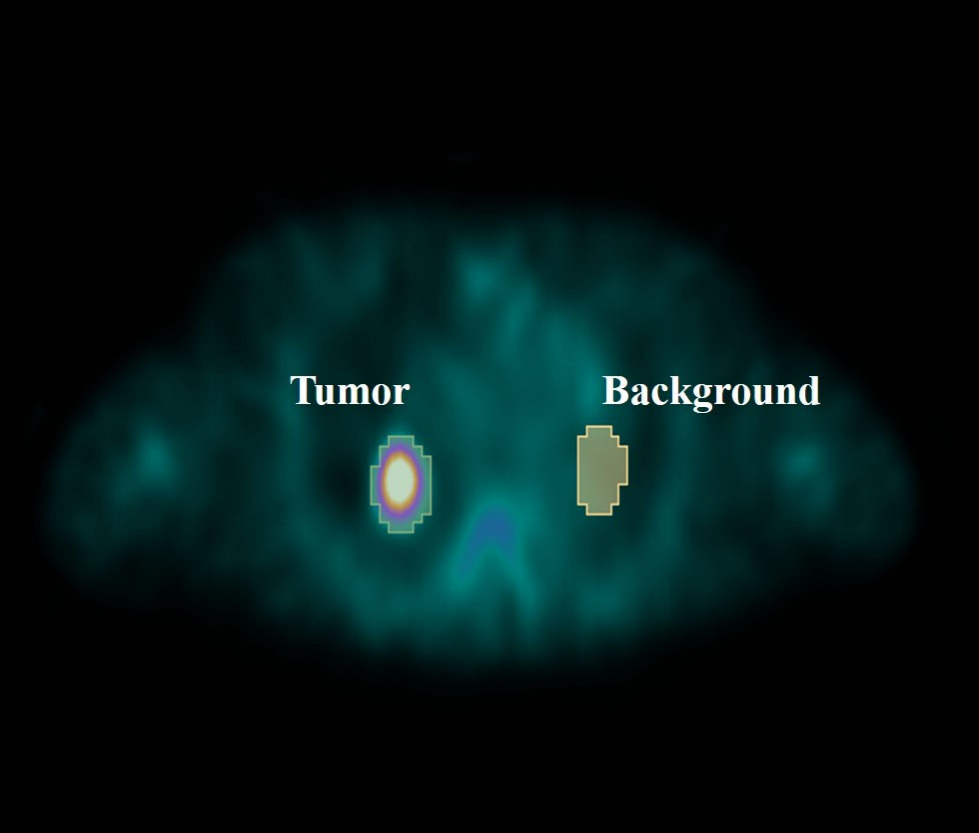
^99m^Tc-3PRGD_2_ chest SPECT images of the same patient based on NAC, CT-AC, and DL-AC. The top row are SPECT axial images (NAC, CT-AC, and DL-AC), and the bottom row are SPECT coronal images (NAC, CT-AC, and DL-AC). It can be seen that DL-AC significantly improves the contrast of the chest image, and the corrected image is close to the CT-AC image. On the axial image, the maximum diameter of the patient’s lung lesion was 49.98 mm, and the maximum T/B of the lesion on NAC, CT-AC, and DL-AC were 2.91, 3.80, and 3.78, respectively.

**Figure 4:**
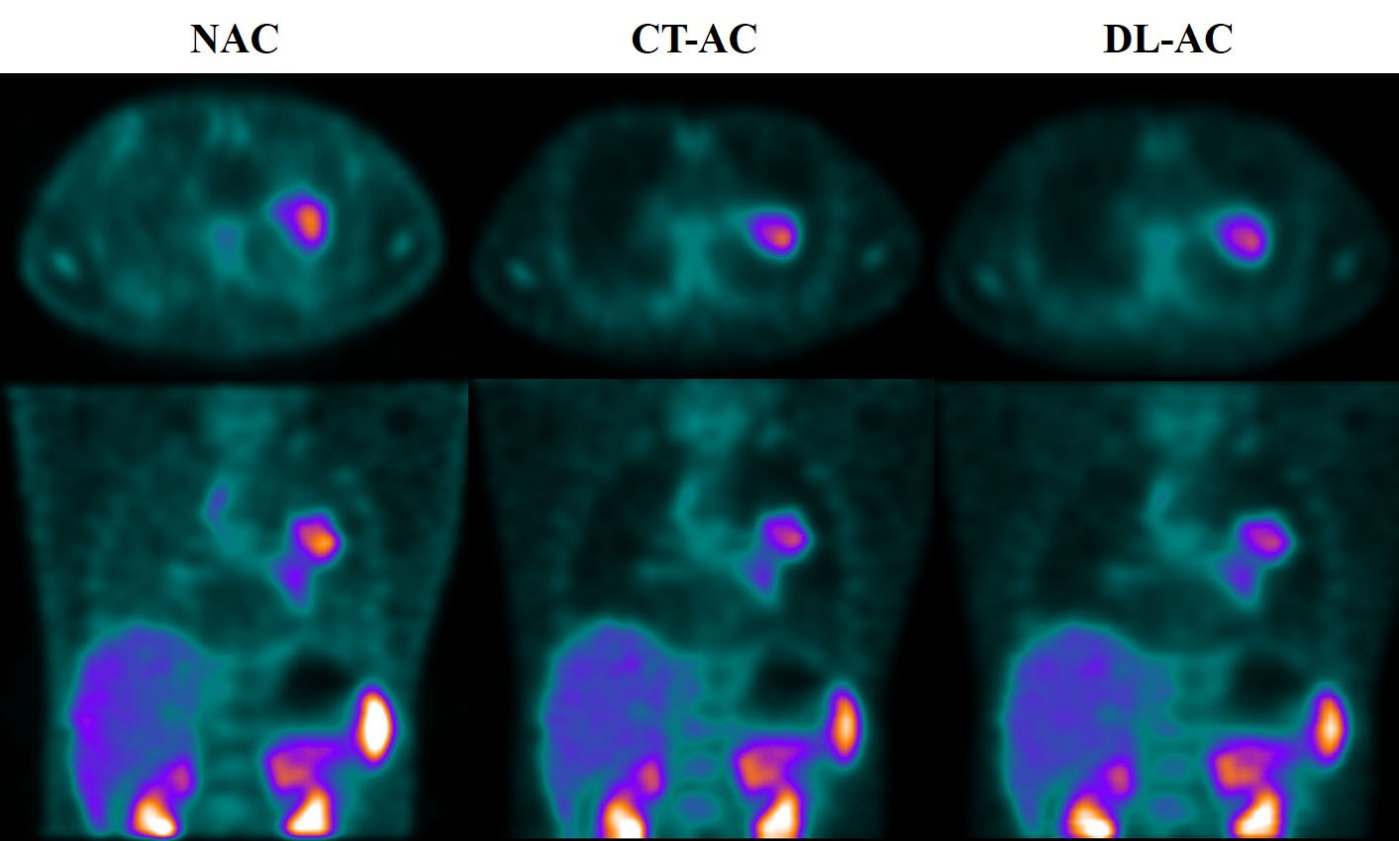
The error image between CT-AC and DL-AC in the SPECT axial image of another patient. The green color indicates that the error is 0, and the red color indicates that the error is maximum.

**Table 1** shows that five testing set SPECT imaging quality metrics between DL-AC and CT-AC including mean absolute error (MAE), mean square error (MSE), peak signal-to-noise ratio (PSNR), structural similarity (SSIM), normalized root mean square error (NRMSE), and normalized mutual information (NMI) is 2.62 ± 0.45, 58.5 ± 14.85, 45.67 ± 2.80, 0.82 ± 0.02, 0.07 ± 0.04, and 1.58 ± 0.06, respectively. These results indicate that MAE < 3.50, MSE < 65.00, PSNR > 42.00, SSIM > 0.80, NRMSE < 0.11, and NMI > 1.60 (mean ± SD). **Figure 3** illustrates ^99m^Tc-3PRGD_2_ chest SPECT images of the same patient based on NAC, DL-AC, and DL-AC. The top row is SPECT axial image (NAC, CT-AC, and DL-AC), and the bottom row is SPECT coronal image (NAC, CT-AC, and DL-AC). It shows that DL-AC significantly improved the contrast of the chest image, and the SPECT images of DL-AC is close to those of CT-AC. **Figure 4** shows the error between CT-AC and DL-AC in SPECT axial image of another patient. In **Figure 4**, green indicates that the error is 0, and red indicates that the error is maximum. The error between the two correction methods (DL-AC and CT-AC) at the lesion site is very small. Outside the chest lesion, the error is close to 0 (green).

**Table 1:** Five testing set SPECT imaging quality metrics between DL-AC and CT-AC.

case	MAE^*^	MSE^*^	PSNR^*^	SSIM^*^	NRSME^*^	NMI^*^
1	2.55	63.74	49.11	0.81	0.06	1.63
2	1.88	34.54	47.51	0.84	0.07	1.64
3	3.06	75.15	45.99	0.83	0.01	1.58
4	2.76	60.20	43.26	0.84	0.08	1.54
5	2.85	58.89	42.49	0.80	0.09	1.50
Mean ± SD	2.62 ± 0.45	58.50 ± 14.85	45.67 ± 2.80	0.82 ± 0.02	0.07 ± 0.04	1.58 ± 0.06

*MAE, mean absolute error; MSE, mean-square error; PSNR, Peak signal-to-noise ratio; SSIM, structural similarity; NRMSE, normalized root mean square error; NMI, normalized mutual information

In order to analyze the differences between the two methods more precisely, a quantitative analysis was performed. The ^99m^Tc-3PRGD_2_ chest SPECT lesion T/B in both SPECT CT-AC and DL-AC is shown in **Table 2**, which shows that the lesions T/B (maximum) between CT-AC and DL-AC in the training and testing sets were almost the same (3.78 ± 2.07 vs. 3.79 ± 2.07; 4.36 ± 3.52 vs. 4.33 ± 3.09, p > 0.5), with no difference. The T/B (mean) between DL-AC and CT-AC in the training and testing sets is also not significantly different (3.41 ± 2.07 vs. 3.36 ± 1.77; 2.73 ± 1.36 vs. 2.86 ± 1.48, p > 0.5). The axial image in **Figure 3** shows that the maximum diameter of the patient’s lung lesion is 49.98 mm, and the maximum T/B of the lesion on NAC, CT-AC, and DL-AC are 2.91, 3.80, and 3.78, respectively. DL-AC obtained lesion T/B close to CT-AC.

**Table 2:** Comparison of T/B between CT-AC and DL-AC on chest SPECT in 53 patients.

Variable	CT-AC	DL-AC	Statistics	P-value
Training set (n = 48)				
T/B maximum	3.78 ± 2.07	3.79 ± 2.07	-0.017	0.987
T/B mean	3.41 ± 2.07	3.36 ± 1.77	0.625	0.534
Testing set (n = 5)				
T/B maximum	4.36 ± 3.52	4.33 ± 3.09	0.015	0.988
T/B mean	2.73 ± 1.36	2.86 ± 1.48	-0.252	0.807
Total data set (n = 53)				
T/B maximum	3.84 ± 2.20	3.84 ± 2.15	0.000	0.994
T/B mean	3.34 ± 2.02	3.40 ± 1.73	0.551	0.583

* T/B, tumor-to-background; CT-AC, CT-attenuation correction; DL-AC, deep learning-attenuation correction.

## Discussion

Attenuation correction is particularly important for accurate quantitative analysis of SPECT images (9–11), which are the basis for quantitative disease analysis, therapeutic efficacy evaluation, and pharmacokinetic studies. From the early uniformity attenuation correction to CT-AC and then to the latest deep learning-based methods, attenuation correction has significantly improved the image quality and accuracy of SPECT. This study shows that in the testing set of ^99m^Tc-3PRGD_2_ chest SPECT images, the imaging quality metrics between DL-AC and CT-AC indicates that PSNR > 42, SSIM > 0.8, and NRMSE < 0.11. Lung lesions T/B in the training and testing sets do not differ between CT-AC and DL-AC (p > 0.5). Overall, it is superior to the reported results (6, 7).

Using deep learning to correct the attenuation in chest images of lung cancer patients can significantly improve the image quality. The absolute error is <4, the similarity is >0.80, and PSNR is >42. In comparison with the study of Yang et al. on cardiac DL-AC, the present study was superior to the results of Yang et al. on NRMSE (0.07 ± 0.04 vs. 0.148 ± 0.095) and PSNR (45.67 ± 2.80 vs. 36.20 ± 4.10), but slightly lower on SSIM (0.82 ± 0.02 vs. 0. 993 ± 0.006). The PSNR is significantly higher than that of Yang and other reported studies, which may be due to the high uptake of ^99m^Tc-3PRGD_2_ targeting drug lesions and low uptake of non-target tissues. Due to the different radiopharmaceuticals used in the two studies, there are significant differences in the distribution of the drugs in the chest and heart. SSIM is relatively low, this may be due to the fact that the study of Yang et al. on DL-AC selected the small-field cardiac images as the dataset, whereas the chest anatomy in this study was more complex, and the image field was larger. These results indicate that the DL-AC method can meet clinical needs and can be used as a method to correct the ^99m^Tc-3PRGD_2_ chest SPECT attenuation in lung cancer patients.

This study is employed based on MONAI, 3D Unet framework because MONAI is a deep learning platform built for medical imaging. For the choice of neural network, the Unet framework in 3D rather than 2D was used in this study. This is mainly due to the fact that the chest SPECT image is a 3D structure after reconstruction, and the 2D approach tends to lead to interlayer errors (12). Torkaman et al. used Conditional Generative Adversarial Networks (CGAN) corrected for myocardial attenuation, and the preliminary results showed no significant advantage (13). This study provides a new method for accurate attenuation correction of SPECT devices not equipped with co-location CT and has clinical practical application value.

There are also some limitations in this study. The DL-AC model was established for Philips equipment, and the model of Siemens and GE equipment is also needed in the future. In addition, the current model group data set is small, which needs to be increased.

## Conclusion

Our preliminary research results indicate that using DL-AC method to directly correct ^99m^Tc-3PRGD_2_ chest SPECT images is highly accurate and feasible for SPECT without configuration with CT or treatment effect evaluation using multiple SPECT/CT scans.

## Data availability statement

The raw data supporting the conclusions of this article will be made available by the authors, without undue reservation.

## Ethics statement

The studies involving human participants were reviewed and approved by the ethics committee of Chinese Academy of Medical Sciences and Peking Union Medical College Hospital. The patients/participants provided their written informed consent to participate in this study.

## Author contributions

All authors contributed to conception and design of the study. HX, TW, XJ, and JT completed data collection, data processing, and analysis. XJ and JT completed illustration preparation, and HX and TW completed thesis writing. JB, HJ, and FL guided the research design, data verification, and paper revision. All authors contributed to the article and approved the submitted version.
